# Supplementary material for: N-(3-oxododecanoyl)-L-homoserine lactone interactions in the breast tumor microenvironment: Implications for breast cancer viability and proliferation in vitro
Source: PLoS One. 2017 Jul 10;12(7):e0180372. doi: 10.1371/journal.pone.0180372 (PMC5503244; doi:10.1371/journal.pone.0180372)
Supplement: S1 Fig — (PDF) [file pone.0180372.s001.pdf]

Proliferation assay image analysis

The semi-quantitative analysis used to analyze the EdU and DAPI stained nuclei from the proliferation assay is depicted in S1 Fig. The 8-bit grayscale confocal image stacks (stacks of images from the same plate with the same stain) were imported into ImageJ. With the first 0 µM OdDHL image (control) selected, the images were first subjected to the integrated 'Subtract Background' algorithm in ImageJ. Next, the integrated 'Threshold' algorithm was then applied to the images and adjusted in order to fully show all nuclei without introducing noise into the image (threshold set just before noise appeared in image). Lastly, the integrated 'Analyze Particles' algorithm was applied to the image and the area covered by stained nuclei was calculated (denoted as "%Area"). After all areas were found using this method, the percent area of the EdU-tagged nuclei was divided by the percent area of the DAPI-tagged (all) nuclei. The fraction calculated from the ratio of the percent areas was converted to a percentage and considered the percent proliferation for the two hour incubation period at each condition.

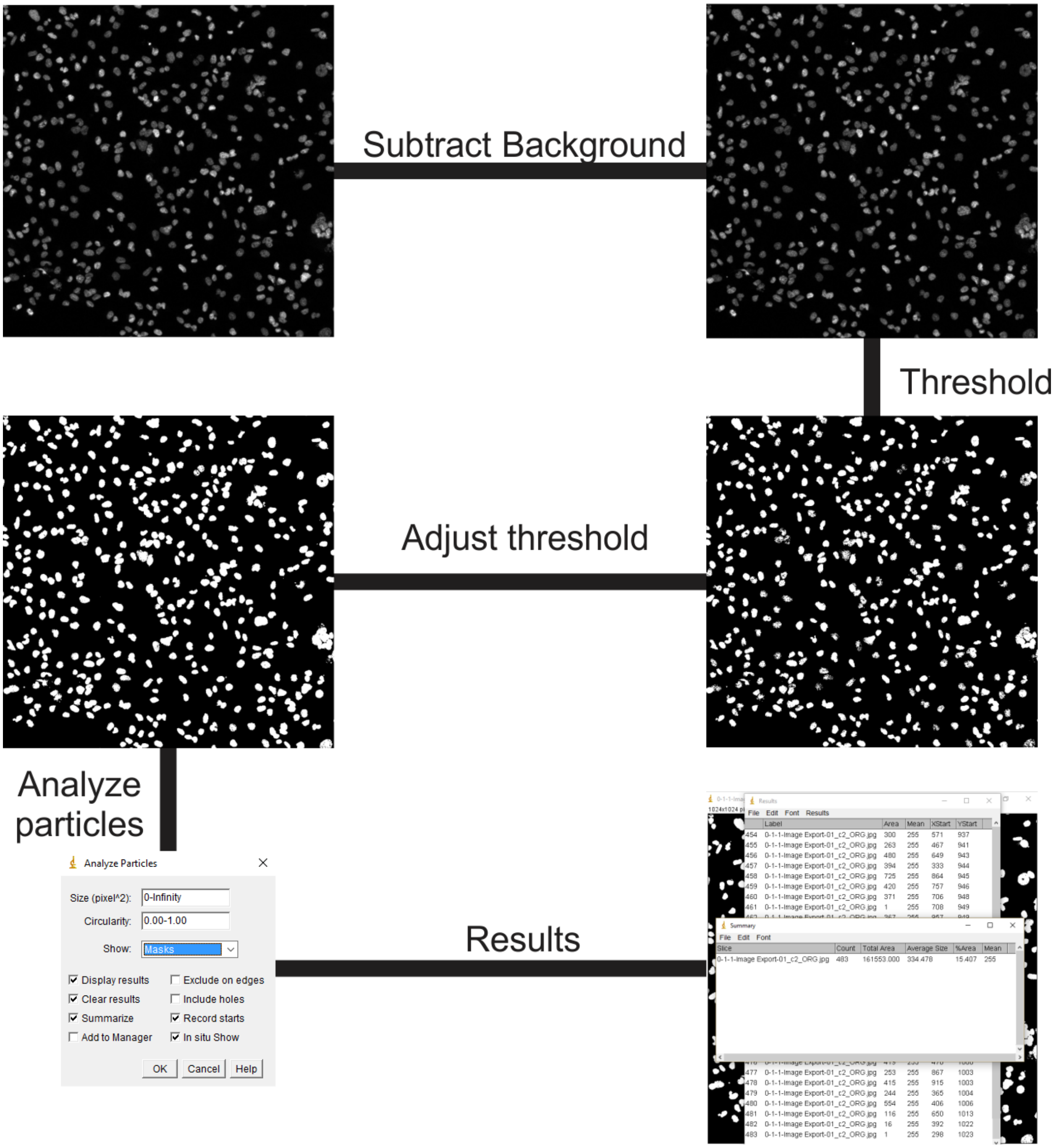

S1 Fig. Diagram of the image analysis method in ImageJ.

After importing the 8-bit grayscale images into ImageJ, the background was subtracted, a threshold was applied and adjusted, and the percent areas of nuclei coverage was calculated using the integrated 'Analyze Particles' algorithm.
