# Supplementary material for: N-(3-oxododecanoyl)-L-homoserine lactone interactions in the breast tumor microenvironment: Implications for breast cancer viability and proliferation in vitro
Source: PLoS One. 2017 Jul 10;12(7):e0180372. doi: 10.1371/journal.pone.0180372 (PMC5503244; doi:10.1371/journal.pone.0180372)
Supplement: S2 Fig — (PDF) [file pone.0180372.s002.pdf]

Apoptosis/necrosis assay image analysis

The semi-quantitative analysis used to analyze the annexin V-FITC and propidium iodide stained cells from the apoptosis/necrosis assay is depicted below in S2 Fig. The original 8-bit grayscale confocal image stacks (from the same plate with the same stain) were imported into ImageJ. The integrated 'Measure' algorithm was applied to each image in order to find the "Mean Gray Value" or MGV (preselected with "Set Measurements" under "Analyze"). After all MGVs were found using this method, the average MGV for each condition was calculated and all MGVs were normalized to the MGV for appropriate 0.6% DMSO control. The fraction calculated from the ratio of the MGVs was converted into a percentage to conduct a statistical analysis of percent increase of apoptosis and necrosis associated with 400 µM OdDHL compared to the 0.6% DMSO control.

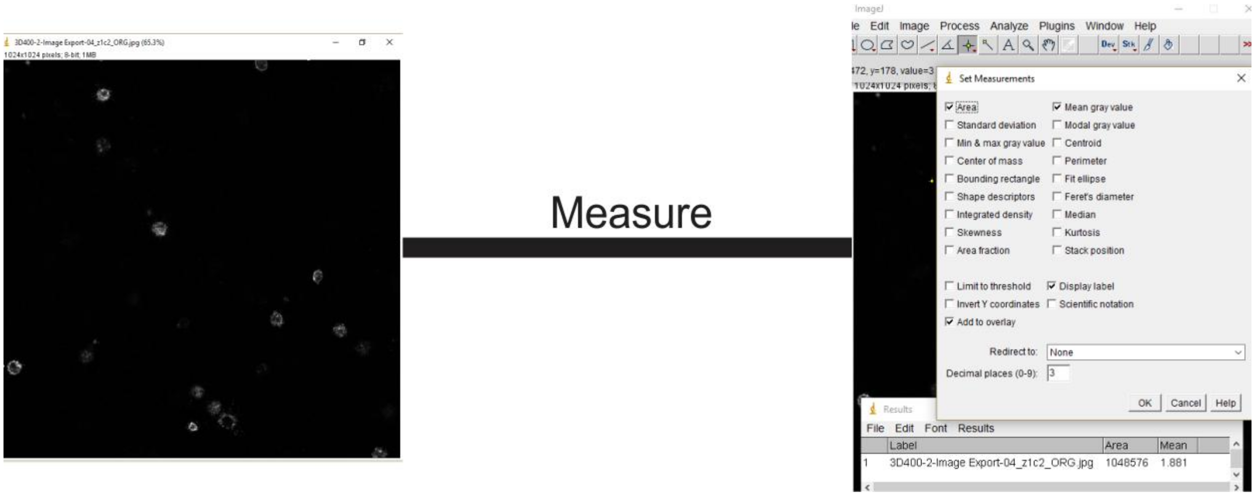

S2 Fig. Diagram of the image analysis method in ImageJ.

After importing the 8-bit grayscale images into ImageJ, the MGV was measured and used as a relative metric of annexin V-FITC and propidium iodide expression.
